# An efficient simulation for quantum secure multiparty computation

**DOI:** 10.1038/s41598-021-81799-z

**Published:** 2021-01-26

**Authors:** Kartick Sutradhar, Hari Om

**Affiliations:** Department of Computer Science and Engineering, Indian Institute of Technology (ISM), Dhanbad, 826004 India

**Keywords:** Computer science, Information technology, Quantum information, Quantum simulation, Qubits

## Abstract

The quantum secure multiparty computation is one of the important properties of secure quantum communication. In this paper, we propose a quantum secure multiparty summation (QSMS) protocol based on (*t*, *n*) threshold approach, which can be used in many complex quantum operations. To make this protocol secure and realistic, we combine both the classical and quantum phenomena. The existing protocols have some security and efficiency issues because they use (*n*, *n*) threshold approach, where all the honest players need to perform the quantum multiparty summation protocol. We however use a (*t*, *n*) threshold approach, where only *t* honest players need to compute the quantum summation protocol. Compared to other protocols our proposed protocol is more cost-effective, realistic, and secure. We also simulate it using the IBM corporation’s online quantum computer, or quantum experience.

## Introduction

In quantum internet, the secure quantum communication is an essential property. The secure quantum communication can be provided by the quantum key distribution (QKD)^1–5^, secure quantum channel^6–9^, dense quantum measurement^10–13^, and quantum secure multiparty summation (QSMS). In quantum computing, the QSMS is a fundamental paradigm for secure quantum communication^14–19^. The QSMS can be used to build many complex protocols^20–31^ like multiplication, sorting^32–35^, voting^36,37^, auction, etc. The QSMS includes a list of secrets ${\mathbb {S}}$ and a set of players ${\mathbb {P}}$. The list of secrets is shared among *n* players ${\mathbb {P}}=\{P_1, P_2, \dots , P_n\}$ over a finite field $\mathbb {F}_d$, where *d* denotes a large prime. The players ${\mathbb {P}}=\{P_1, P_2, \dots , P_n\}$ jointly perform the summation by without disclosing the privacy of their secrets. The security of this protocol is guaranteed until some players reveal their secrets. Suppose, the dealers *A* and *B* contain two secrets *X* and *Y* (for simplicity, we take only two secrets, but the secrets can be any number *n* or more than *n*) and the players ${\mathbb {P}}=\{P_1, P_2, \dots , P_n\}$ want to compute the secure summation without revealing their secrets. Consider that $X, Y \in {\mathbb {S}}$ are two secrets of the dealers *A* and *B*, respectively. The dealers *A* and *B* share two secrets *X* and *Y* among *n* players ${\mathbb {P}}=\{P_1, P_2, \dots , P_n\}$ using the Shamir’s secret sharing^38^. The $X_1, X_2, \dots ,X_n$ and $Y_1, Y_2, \dots , Y_n$ denote the shares of secrets *X* and *Y*, respectively. The players ${\mathbb {P}}=\{P_1, P_2, \dots , P_n\}$ want to execute $(X_i + Y_i)$, $i=1, 2, \dots , n$, without disclosing their shares. We simulate this protocol by using the IBM quantum computer or quantum experience^39,40^, which is presented at T.J. Watson lab in USA. The novelties of this QSMS can be summarized as follows.

- The proposed protocol is more secure against the participant attack and it has the threshold approach of (*t*, *n*), in which only the *t* honest players can securely compute the multiparty quantum summation.
- Compared to other protocols, the proposed protocol is more realistic and cost-effective.

In secure multiparty classical computation, there exist many summation protocols, but they are unable to provide secure communications; whereas, the QSMS is unconditional secure as it uses the concepts of quantum mechanics. In secure multiparty quantum computation, there have been discussed many summation protocols. In 2002, Heinrich^41^ discussed a QSMS protocol. In 2003, Heinrich^42^ introduced another QSMS protocol with Boolean setting. In 2006, Hillery^43^ discussed a QSMS, based on two-particle entanglement. In 2007, Du et al.^44^ discussed a QSMS protocol based on non-orthogonal states. This protocol’s modulo is $n+1,$ where total number of players is *n*. In 2010, Chen et al.^45^ implemented a QSMS protocol based on multi-particle entanglement with modulo 2. In 2014, Zhang et al.^46^ discussed a QSMS protocol based on polarization of photon with modulo 2. In 2015, Zhang et al.^47^ implemented a quantum summation protocol for three-party with modulo 2. There are some limitations in the above mentioned protocols^44–47^, as discussed below.

- These protocols are based on a threshold approach of (*n*, *n*), where all players need to perform the secure multiparty quantum summation. If any player is rational, then these protocols cannot be executed efficiently.
- These protocols are not cost-efficient because they have bit-by-bit operations.
- These protocols have some security issues because their modulo is too small.

Shi et al.^48^ implemented a QSMS protocol, which can compute the summation efficiently with large modulo *p*, but it has the threshold approach of (*n*, *n*), where $p=2^q$ and *q* is number of qubits. Shi and Zhang^49^ discussed a QSMS protocol, which can compute the summation efficiently, but it is not secure because it has only two-party. Zhang et al.^50^ implemented a QSMS protocol based on quantum secure multiparty computation, but its modulo is 2 only. Liu et al.^51^ discussed a QSMS protocol based on the threshold approach of (*n*, *n*) with modulo 2, and its form of computation is bit-by-bit. In 2018, Yang and Ye^52^ discussed a QSMS protocol with modulo *d*. Its form of computation is secret-by-secret, but it has the threshold approach of (*n*, *n*). In 2019, Jiao et al.^53^ discussed a QSMS protocol, which has the threshold approach of (*n*, *n*), and its form of computation is bit-by-bit. In the same year, Zhang et al.^54^ have discussed a QSMS protocol. Its modulo is *d*, but it has the threshold approach of (*n*, *n*). In 2020, Sutradhar and Om introduced a quantum secret sharing^55^ protocol. This protocol is efficient and has (*t*, *n*) threshold approach, but it has more computational cost because it uses CNOT gate and SHA1. This protocol does not discuss about the realistic implementation, collective and coherent attacks. In the same year, Sutradhar and Om^56^ discussed a multiparty quantum summation protocol. This protocol is efficient and has (*k*, *n*) threshold approach, but it has more computational cost because it uses SUM gate, where *k* denotes the players of the qualified subset. This protocol does discuss about the collective and coherent attacks. Recently, Sutradhar and Om^57^ introduced another quantum protocol for secure multiparty summation. This protocol is efficient and has (*t*, *n*) threshold approach, but it has more computational cost because it uses the SUM gate. This protocol does not discuss about the realistic implementation. Moreover, the proposed protocol is more secure, realistic and cost-effective as compared to the these protocols^55–57^. In this paper, we propose a QSMS protocol with a form of secret-by-secret computation. The proposed protocol has the threshold approach of (*t*, *n*), where only *t* honest players need to execute the secure multiparty quantum summation efficiently and cost-effectively without disclosing their secrets.

## Preliminaries

In this section, we discuss the Shamir’s Secret Sharing (SSS), Pauli operator, and Quantum Fourier Transform (*QFT*).

### Shamir’s secret sharing

The SSS^38^ contains ${\mathbb {P}} = \{P_1, P_2, \dots , P_n\}$, a dealer, and *n* players. It is formed in two phases as discussed below.

#### Secret sharing phase

In this phase, the dealer uses $(t-1)$-degree polynomial *f*(*x*) to share the secret and distribute those shares among *n* players, each player $P_i$ contains only $f(x_i)$, $i=1, 2, \dots , n$.

#### Secret reconstruction phase

In this phase, reconstruction is performed by the threshold number of players using the Lagrange Interpolation, as discussed below.

1 $$\begin{aligned} f(x) = \sum _{u=1}^{t} f(x_u) \prod _{1 \le z \le t, z \ne u} \frac{x - x_z}{x_u - x_z} \end{aligned}$$

For $x=0$, Eq. (1) can be simplified as follows:

2 $$\begin{aligned} \begin{aligned} f(0)&= \sum _{u=1}^{t} f(x_u) \prod _{1 \le z \le t, z \ne u} \frac{ - x_z}{x_u - x_z} \\&= \sum _{u=1}^{t} f(x_u) \prod _{1 \le z \le t, z \ne u} \frac{x_z}{x_z - x_u} \end{aligned} \end{aligned}$$

where $$u,z =1, 2, \dots , t$$.

### Pauli operator

The Pauli operator is defined as follows:

3 $$\begin{aligned} U_{m, 0} = \sum _{c=0}^{d-1} \omega ^{c.0} |c+m\rangle \langle c| \end{aligned}$$

where $$m\in \{0, 1, \dots , d-1 \}$$.

### Quantum Fourier transform (*QFT*)

The *QFT*^58^ is an extension of the regular Fourier discrete transformation. For $v\in \{0, 1, \dots , d-1 \}$, the *QFT* is defined as follows:

4 $$\begin{aligned} QFT |v\rangle = \frac{1}{\sqrt{d}} \sum _{c=0}^{d-1} e^{2\pi i\frac{v}{d}c} |c\rangle . \end{aligned}$$

## Our contribution

In this section, we propose a (t,n) threshold QSMS protocol. Let the dealers *A* and *B* have two secrets (for simplicity, we only take two secrets but the secrets can be any number *n* or more than *n*, where *n* denotes total no of players) *X* and *Y*, respectively, and *n* players want to jointly perform the summation $(S=X+Y)$ without revealing their secrets. In this protocol, each qualified subset ${\mathbb {P}}=\{P_1, P_2, \dots , P_t\}$ contains a $k^{th}$ player as an initiator. We assume that $k^{th}$ player is $P_1$, which acts as an initiator. The initiator $P_1$ only contains his share value, nothing else. The process of quantum secure multiparty summation is given as follows.

- **Step 1:** *A* and *B* choose two distinct $(t-1)$-degree polynomials $f(x)=X + \alpha _1x + \alpha _2x^2 + \dots + \alpha _{t-1}x^{t-1}$ and $g(x)=Y + \beta _1x + \beta _2x^2 + \dots + \beta _{t-1}x^{t-1}$, *X* and *Y* are secrets and the symbol $'+'$ is defined as addition modulo *d*, *d* is a prime such that $n \le d \le 2n$. The *A* and *B* use the Shamir’s secret sharing to compute the shares $f(x_i)$ and $g(x_i)$, respectively, which are distributed among *n* players using an authenticated classical channel. The player $P_i$ only knows the shares $f(x_i)$ and $g(x_i)$, $i= 1, 2, \dots , n$.
- **Step 2:** Player $P_i$ computes $h(x_i)= f(x_i) + g(x_i)$, $i= 1, 2, \dots , n,$ and possesses the share $h(x_i)$ only.
- **Step 3:** Player $P_u$ computes the shadow $(m_u)$ of the share $h(x_u)$, $u= 1, 2, \dots , t$, as follows.
  5 $$\begin{aligned} m_u = h(x_u) \prod _{1\le z\le t, z\ne u} \frac{x_z}{x_z - x_u} \mod d \end{aligned}$$
- **Step 4:** Initiator player $P_1$ prepares $t-$particle entangled states as follows.
  6 $$\begin{aligned} |\Psi _1\rangle = \frac{1}{\sqrt{d}} \sum _{c=0}^{d-1} |c\rangle _1 |c\rangle _2 \dots |c\rangle _t \end{aligned}$$
  Player $P_1$ sends the particle $|c\rangle _u$ to player $P_u$, $u=2, 3, \dots , t$.
- **Step 5:** Each player $P_u$ performs the *QFT*^52^ on his particle $|c\rangle _u$ as follows:
  7 $$\begin{aligned} QFT |c\rangle _1 = \frac{1}{\sqrt{d}} \sum _{a_1=0}^{d-1} e^{2\pi i\frac{c}{d}a_1} |a_1\rangle . \end{aligned}$$
  Each player $P_u$, $(u=1, 2, \dots , t)$, also applies the Pauli operator $U_{m_u, 0}$ on his particle as follows:
  8 $$\begin{aligned} U_{m_1, 0} = \sum _{c=0}^{d-1} \omega ^{c.0} |c+m_1\rangle \langle c| \end{aligned}$$
  After performing the *QFT* and Pauli operator, the resultant state $|\Psi _2\rangle$ is obtained as follows.
  9 $$\begin{aligned} \begin{aligned} |\Psi _2\rangle&= U_{m_1, 0} QFT \otimes U_{m_2, 0} QFT \otimes \dots \otimes U_{m_t, 0} QFT |\Psi _1\rangle \\&= d^{-\frac{t+1}{2}} \sum _{0 \le a_1, \dots , a_t <d,~ a_1 + ,\dots , + a_t = 0 \mod ~d} |a_1+m_1\rangle |a_2+m_2\rangle \dots |a_u+m_u\rangle \end{aligned} \end{aligned}$$
  **Step 6:** Each player $P_u$ performs the measurement operation on his particle $|a_u+m_u\rangle$ in computational basis $\{ |1\rangle , |2\rangle , \dots , |d-1\rangle \}$, and broadcasts his measurement results $a_u+m_u$, where $u=1, 2, \dots , t$.
- **Step 7:** Finally, the players in qualified subset calculate the summation jointly by summing their results of measurement: $S=\sum _{u=1}^{t} a_u+m_u \mod ~d$.

## Correctness

### Lemma 1

*If QFT and Pauli operators are honestly performed by all players in a qualified subset* ${\mathbb {P}}=\{P_1, P_2, \dots , P_t\}$, *then they can jointly compute the multiparty quantum summation* $(\sum _{u=1}^{t} m_u \mod ~d)$ *correctly*.

### Proof

If QFT and Pauli operators are honestly performed by every player in the qualified subset ${\mathbb {P}}=\{P_1, P_2, \dots , P_t\}$, the quantum state is obtained as follows.

10 $$\begin{aligned} \begin{aligned} |\Psi _2\rangle&= U_{m_1, 0} QFT \otimes \dots \otimes U_{m_t, 0} QFT \Big (\frac{1}{\sqrt{d}} \sum _{c=0}^{d-1} |c\rangle _1 \dots |c\rangle _t \Big )\\&=\frac{1}{\sqrt{d}} \sum _{c=0}^{d-1} U_{m_1, 0} QFT |c\rangle _1 \otimes \dots \otimes U_{m_t, 0} QFT |c\rangle _t\\&=\frac{1}{\sqrt{d}} \sum _{c=0}^{d-1} \Big ( U_{m_1, 0} \frac{1}{\sqrt{d}} \sum _{a_1=0}^{d-1} \omega ^{a_1c} |a_1\rangle \Big ) \otimes \dots \otimes \Big ( U_{m_t, 0} \frac{1}{\sqrt{d}} \sum _{a_1=0}^{d-1} \omega ^{a_tc} |a_t\rangle \Big )\\&= d^{-\frac{t+1}{2}} \sum _{0 \le a_1, \dots , a_t<d} \sum _{c=0}^{d-1} \omega ^{(a_1 + \dots + a_t)c} |a_1+m_1\rangle \otimes \dots \otimes |a_t+m_t\rangle \\&= d^{-\frac{t+1}{2}}s_0d\sum _{0 \le a_1, \dots , a_t <d, a_1+ \dots + a_t=0\mod d} |a_1+m_1\rangle \otimes \dots \otimes |a_t+m_t\rangle \end{aligned} \end{aligned}$$

Each player $P_u$, $u=1, 2, \dots , t$, performs the measurement operation on his own particle in computational basis $|a_u+m_u\rangle$. The QSMS can be computed after receiving the measurement results of each player $P_u$, $u=1, 2, \dots , t$. The QSMS of secret can be calculated as follows.

11 $$\begin{aligned} \sum _{u=1}^{t} a_u+m_u \overset{d}{\equiv } \sum _{u=1}^{t} a_u + \sum _{u=1}^{t} m_u \overset{d}{\equiv } \sum _{u=1}^{t} m_u \mod ~d \end{aligned}$$

Thus, the multiparty quantum summation of secrets equals to $\sum _{u=1}^{t} m_u \mod ~d$. $\square$

## Illustration of secure multiparty quantum summation

Here, we use a numerical example to discuss the working of the proposed protocol. Let *A* and *B* hold two secrets 2 and 3, respectively and they want to perform the summation $S=(2+3)$. *A* and *B* choose threshold $(t)=3$, total number of players $(n)=7$, and prime $(d)=11$. Suppose *A* and *B* select two different polynomials $f(x)=2 + x + x^2 \mod ~11$ and $g(x)=3 + x + x^2 \mod ~11$, respectively. They calculate the shares $f(x_i)$ and $g(x_i), i=1,2,\dots ,7$ using the Shamir’s secret sharing, and allocate these shares to 7 players. Each player $P_i, i = 1, 2, \dots , 7$, performs $h(x_i)= f(x_i) + g(x_i) \mod ~11$. The calculation of shares $h(x_i)$ is shown in Table 1. Each player $P_u$, u= 1, 2, 3, computes the shadow of the shares $m_u$, as $m_1 = 9. \Big (\frac{2}{2 - 1} . \frac{3}{3 - 1} \Big ) \mod 11 =5$, $m_2 = 6. \Big (\frac{1}{1 - 2} . \frac{3}{3 - 2} \Big ) \mod 11 = 4$, and $m_3 = 7. \Big (\frac{1}{1 - 3} . \frac{2}{2 - 3} \Big ) \mod 11 =7$, respectively (using Eq. 5). The player $P_1$ now computes $|\Psi _1\rangle = \frac{1}{\sqrt{11}} \sum _{c=0}^{10} |c\rangle _1 |c\rangle _2 |c\rangle _3$ and sends the particle $|c\rangle _u$ to player $P_u, u=2, 3$. Each player $P_u, u=1, 2, 3,$ applies the *QFT* and Pauli operator $U_{5, 0}$, $U_{4, 0}$, $U_{7, 0}$ on his particle, respectively, (as per Eq. 9).

**Table 1:** Share computation.

Players		$P_1$	$P_2$	$P_3$	$P_4$	$P_5$	$P_6$	$P_7$
Shares	$f(x_i)$	4	8	3	0	10	0	3
	$g(x_i)$	5	9	4	1	0	1	4
	$h(x_i)$	9	6	7	1	10	1	7

12 $$\begin{aligned} \begin{aligned} |\Psi _2\rangle&= U_{5, 0} QFT \otimes U_{4, 0} QFT \otimes U_{7, 0} QFT \Big (\frac{1}{\sqrt{11}} \sum _{c=0}^{10} |c\rangle _1 |c\rangle _2 |c\rangle _3 \Big )\\&=\frac{1}{\sqrt{11}} \sum _{c=0}^{10} U_{5, 0} QFT |c\rangle _1 \otimes U_{4, 0} QFT |c\rangle _2 \otimes U_{7, 0} QFT |c\rangle _3\\&= 11r_1 \sum _{0 \le a_1, a_2, a_3 <10,~ a_1 + a_2 + a_3 = 0 \mod ~11} |a_1+5\rangle |a_2+4\rangle |a_3+7\rangle \end{aligned} \end{aligned}$$

Each player $P_u, u = 1,2,3,$ performs the measurement operation in computational basis on his particle. The players $P_1$, $P_2$, and $P_3$ broadcast the measurement results $a_1+5$, $a_2+4$, and $a_3+7$, respectively. Finally, they get the summation by summing the results of measurement as follows:

$$ a_1+5+a_2+4+a_3+7 \overset{11}{\equiv } a_1+a_2+a_3+16\overset{11}{\equiv } 16 \mod 11=5. $$

## Simulation results

**Figure 1:**
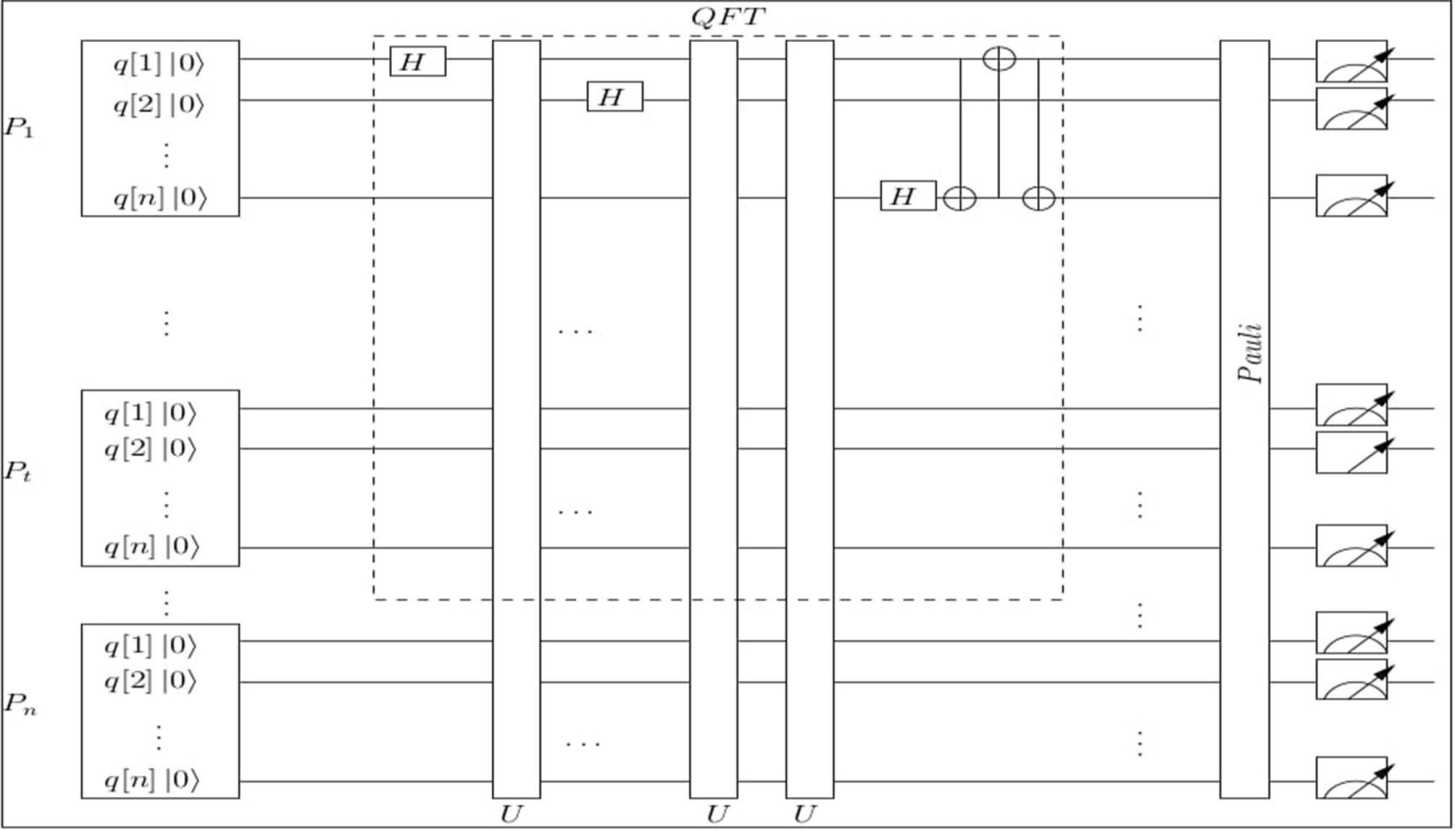
Circuit diagram of QSMS.

We simulate the proposed protocol using the IBM real quantum processor^39,40^, which is available at T.J.Watson lab, USA. We explain the circuit diagram (refer Fig. 1) of our QSMS protocol. The Hadamard gate is taken as the *QFT* in this circuit diagram of QSMS. On his particle, the player $P_u$ applies the *QFT* and also performs the Pauli operator on his particle. Then, each player $P_u$ performs measurement operations on his own particle, and broadcasts the measurement result. Finally, by summing their measurement results, the players jointly calculate the QSMS. The privacy of this protocol is guaranteed until a certain number of players disclose their shares.

We have simulated this circuit of QSMS with 3 players, 5 qubits, and 8192 number of average shots. Initially, the player $P_u$, $u=1,2,3$ performs the *QFT* on his particle $|c\rangle _u$ and also executes the Pauli operator on particle $|c\rangle _u$. Then, each player $P_u, u = 1,2,3,$ executes the measurement operation in computational basis on his particle. The players $P_1$, $P_2$, and $P_3$ broadcast the measurement results $a_1+5$, $a_2+4$, and $a_3+7$, respectively. Finally, they get the summation of 2 and 3 by adding the measurement results as follows:

$$ a_1+5+a_2+4+a_3+7=16 \mod 11=5. $$

**Figure 2:**
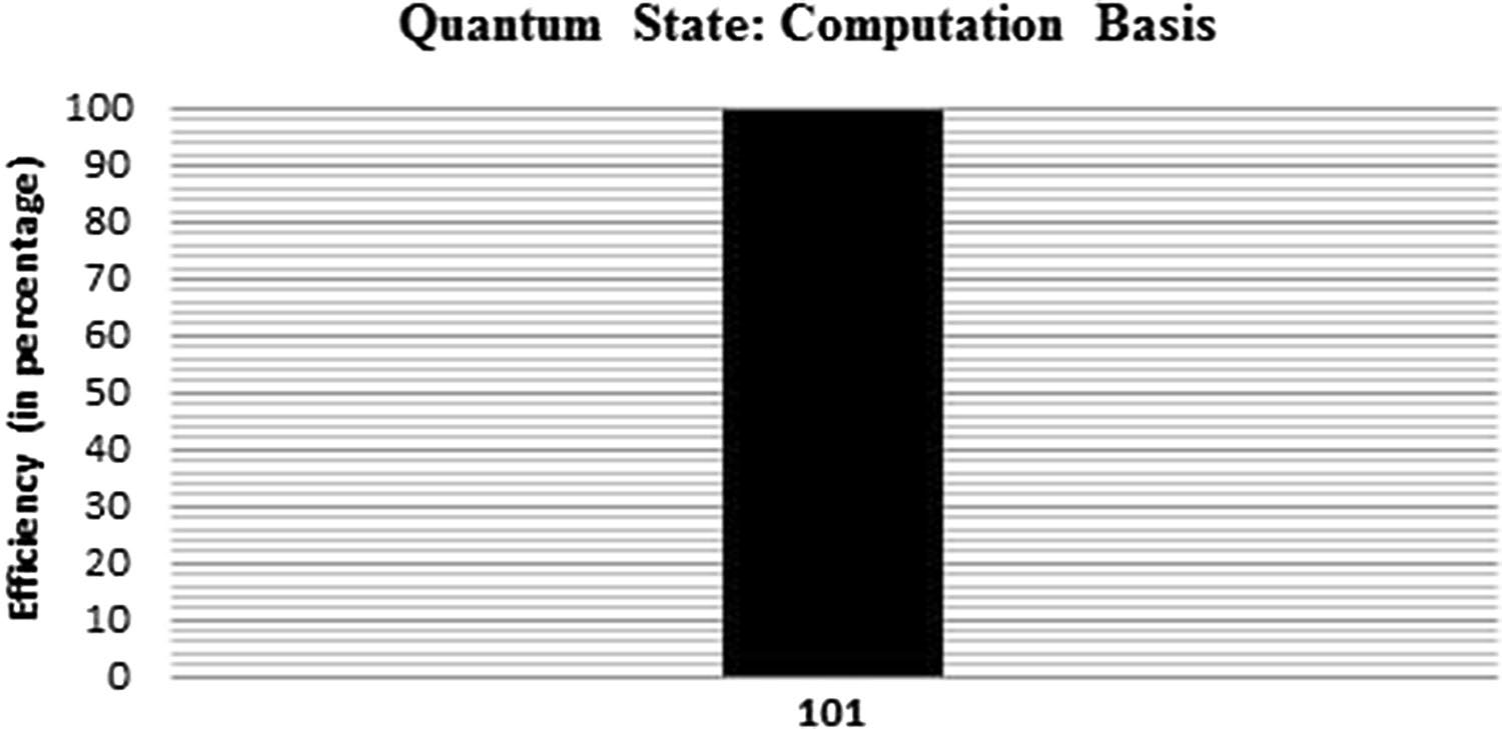
Simulation result.

The simulation result of the proposed summation protocol for 3 players, 5 qubits, and 8192 number of average shots. The state 101 (i.e., binary representation of 5) is calculated efficiently. The result of this simulation using the IBM real quantum processor is shown in Fig. 2.

## Discussion

Here, we address the security and performance analysis based on some properties of the proposed QSMS protocol.

### Security analysis

In this section, we analyze the security of QSMS protocol based on the intercept-resend, entangle-measure, intercept, collective, coherent, and collusion attacks.

*Intercept-resend attack* Suppose an attacker Mallory intercepts the particle $|c\rangle _u$. It measures the quantum particle $|c\rangle _u$ in the computational basis to get the useful data about the share’s shadow ($m_u$). Mallory produces the clone quantum particle $|\bar{c}\rangle _u$ and resends this clone particle to player $P_u$, $u=2,3,\dots t$. If Mallory applies this method to attack, then it can get *c* accurately with probability $\frac{1}{d}$. But, from this attack, Mallory cannot get any useful data about the share’s shadow $m_u$, because the intercepted particle $|c\rangle _u$ does not contain any useful data about the share’s shadow $m_u$.

*Entangle-Measure attack* After the intercept attack, Mallory performs the complex entangle-measure attack on the entangled quantum particle $|c\rangle _u$. In this attack, Mallory performs the measurement operation on the intercepted entangled quantum particle $|c\rangle _u$ in the computational basis to get the useful data about the share’s shadow $m_u$. If Mallory applies the entangle-measure attack, then it can get *c* accurately with probability $\frac{1}{d}$. But, from this attack, Mallory cannot get useful data about the share’s shadow $m_u$, because the intercepted entangled quantum particle $|c\rangle _u$ does not contain any useful data about the share’s shadow $m_u$.

*Intercept attack* Suppose Mallory intercepts the particle $|c\rangle _u$ and measures the quantum particle $|c\rangle _u$ in the computational basis to reveal the useful data about the share’s shadow $m_u$. If Mallory measures the quantum particle $|c\rangle _u$ in the computational basis, then it can get *c* correctly with probability $\frac{1}{d}$. But, from the measurement result *c*, it cannot get any useful data about the share’s shadow $m_u$, because the intercepted particle $|c\rangle _u$ does not carry any useful data about the share’s shadow $m_u$.

*Collective attack* In a collective attack, Mallory prepares an autonomous ancillary particle to communicate with each qudit to get the shadow of share and they perform the joint measurement operation on every ancillary qudit. Suppose Mallory communicates with every qudit of all players by preparing an autonomous ancillary particle $|e\rangle$. After successful interaction, Mallory gets the particle $|o\rangle _x$. Then, Mallory wants to know the shadow of share by performing a computational basis $\{ |1\rangle , |2\rangle , \dots , |d-1\rangle \}$ joint measurement operation. Mallory cannot get any useful data about the share’s shadow from this joint measurement operation because $|o\rangle _x$ does not contain any useful data about the share’s shadow.

*Coherent attack* In this attack, Mallory prepares an autonomous ancillary particle $|c\rangle$ to communicate with the qudits of each player. After interacting, Mallory gets each player’s particle $|o\rangle _x$ and performs a joint measurement operation on all players particle *c* in computational basis $\{ |1\rangle , |2\rangle , \dots , |d-1\rangle \}$. Mallory only gets *o* from the joint measurement result of particle $|o\rangle _x$ with probability $\frac{1}{d}$. But, the joint measurement result *o* does not contain any useful data about the share’s shadow. From this attack, Mallory only gets the interacting particle $|o\rangle _x$, but it cannot learn any useful data about the share’s shadow.

*Collusion attack* In this protocol, each player $P_u$ performs the measurement on his own particle $|a_u+m_u\rangle$ and broadcasts his result of the measurement $a_u+m_u$, $u=1, 2, \dots , t$. From this broadcast, other players cannot get any useful data about the share’s shadow $m_u$. If some rational players $P_{l-1}$ and $P_{l+1}$ jointly want to get the data about the share’s shadow but they cannot get any useful data about the share’s shadow $m_u$ because the initiator $P_1$ transmits only particles $|c\rangle _u$ to all other players and unfortunately $|c\rangle _u$ does not contain any useful data about the share’s shadow $m_u$.

### Performance analysis

We analyze and compare the performance of the proposed (*t*, *n*) threshold summation protocol with the existing summation protocols^44–54^. The protocols^44–47^ are multiparty, but they have the threshold approach of (*n*, *n*) and their type of computation is bit-by-bit. The protocol^48^ is multiparty and its type of computation is secret-by-secret, but it is based on the threshold approach of (*n*, *n*). The protocols^49,50^ perform bit-by-bit computation, but they are based on the threshold approach of (*n*, *n*). The protocol^51^ is multiparty, but its type of computation is bit-by-bit and it has the threshold approach of (*n*, *n*) with modulo is 2. The protocol^52^ is multiparty and its type of computation is secret-by-secret, but it is based on the threshold approach of (*n*, *n*). The protocol^53^ is based on quantum multiparty computation, but its type of computation is bit-by-bit and it has the threshold approach of (*n*, *n*). The protocol^54^ is multiparty and its type of computation is secret-by-secret, but it has the threshold approach of (*n*, *n*), where all honest players need to perform the multiparty quantum summation. This protocol cannot be performed correctly if any player is dishonest. However, our proposed protocol has the threshold approach of (*t*, *n*), in which only honest players of *t* can securely compute the multiparty quantum summation with modulo *d*. In addition, the proposed protocol has secret-by-secret computation type. This protocol can be performed correctly if any *t* players are honest. So, Compared to other protocols, our proposed protocol is more cost-effective, efficient, realistic, and secure, as shown in Table 2. In this table, *Com*., *Comm*., *UO*, *Part*., *MO*, *QFT*, $QFT^{-1}$, *DP*, *EM*, *INCPT*, *MP*, *COLL*, *COL*, *COH*, *IR*, $sec-by-sec$, *Y*, *N*, *MD*, and *CT* denote Computation, Communication, Unitary Operation, Participant, Measure Operation, Quantum Fourier Transform, Inverse Quantum Fourier Transform, Decoy Particle, Entangle-Measure, Intercept, Message Particle, Collective, Collusion, Coherent, Intercept-Resend, secret-by-secret, Yes, No, Modulo, and type of Computation, respectively.

**Table 2:** Comparison with ten protocols.

Protocols	Performance parameters															
	Costs						Attacks						Universality			
	*Com*.				*Comm*.		Outside			*Part*.			Model	MD	Qubit	*CT*
	QFT	$QFT^{-1}$	*MO*	*UO*	*MP*	*DP*	*IR*	*EM*	*INCPT*	*COLL*	*COH*	*COL*				
Ref.^44^	−	−	−	−	−	−	−	−	*N*	−	−	−	(*n*, *n*)	$n+1$	−	Bit-by-bit
Ref.^45^	−	−	−	1	−	−	*Y*	−	*N*	−	−	*Y*	(*n*, *n*)	2	−	Bit-by-bit
Ref.^46^	−	−	−	1	−	−	*Y*	*Y*	*N*	−	−	*N*	(*n*, *n*)	2	−	Bit-by-bit
Ref.^47^	−	−	−	1	−	−	*Y*	−	*N*	−	−	*N*	(*n*, *n*)	2	−	Bit-by-bit
Ref.^48^	1	1	2	$n-1$	−	*n*	*Y*	*Y*	*N*	−	−	−	(*n*, *n*)	*p*	$\lceil \log _{2}^{d} \rceil n$	Sec-by-sec
Ref.^49^	−	−	−	−	−	−	−	−	*N*	−	−	−	(*n*, *n*)	−	$2\lceil \log _{2}^{d} \rceil n$	Bit-by-bit
Ref.^50^	−	−	*n*	−	−	−	*Y*	−	*N*	−	−	*Y*	(*n*, *n*)	2	−	Bit-by-bit
Ref.^51^	−	−	*n*	−	−	*n*	*Y*	*Y*	*N*	−	−	−	(*n*, *n*)	2	2	Bit-by-bit
Ref.^52^	*n*	−	*n*	−	−	$n-1$	*Y*	*Y*	*N*	−	−	*Y*	(*n*, *n*)	*d*	−	Sec-by-sec
Ref.^53^	−	−	*n*	*n*	−	*n*	*Y*	*Y*	*N*	−	−	−	(*n*, *n*)	*p*	−	Bit-by-bit
Ref.^54^	−	−	*n*	1	−	−	*Y*	*Y*	*N*	−	−	*Y*	(*n*, *n*)	−	−	Sec-by-sec
Proposed	1	−	*t*	$t-1$	$t-1$	−	*Y*	*Y*	*Y*	*Y*	*Y*	*Y*	(*t*, *n*)	*d*	−	Sec-by-sec

## Conclusion

In this paper, we have discussed a secret sharing based (*t*, *n*) threshold QSMS protocol. This protocol can be executed efficiently if any *t* number of players are honest. It is secure and efficient because its type of computation is secret-by-secret and its communication type is linear. It can also compute the QSMS if the total number of secrets is more than the total number of players because the linear secret sharing is used to compute the share of secrets. This QSMS protocol is more realistic as compared to the existing multiparty quantum summation protocols because we have simulated this protocol efficiently using IBM quantum computer that provides efficient result after increasing the number of shots.
